# Acute stress negatively impacts on-task behavior and lecture comprehension

**DOI:** 10.1371/journal.pone.0297711

**Published:** 2024-02-06

**Authors:** Anisa Morava, Ali Shirzad, James Van Riesen, Nader Elshawish, Joshua Ahn, Harry Prapavessis

**Affiliations:** School of Kinesiology, Western University, London, ON, Canada; Federal University of Paraiba, BRAZIL

## Abstract

Acute stress has been shown to disrupt cognitive and learning processes. The present study examined the effects of acute stress on mind wandering during a lecture and subsequent lecture comprehension in young adults. Forty participants were randomized to acute stress induction via the Trier Social Stress Test or rest prior to watching a twenty-minute video lecture with embedded mind wandering probes, followed by a lecture comprehension assessment. Stress responses were assessed via heart rate, blood pressure, salivary cortisol, and state anxiety. Individuals exposed to acute stress endorsed greater mind wandering at the first checkpoint and lower lecture comprehension scores. Moreover, state anxiety post stress was positively associated with mind wandering at the first and second checkpoint and negatively associated with lecture comprehension. Only mind wandering at the third checkpoint was negatively correlated with overall lecture comprehension. Taken together, these data suggest that acute stress, mind wandering, and lecture comprehension are inextricably linked.

## Introduction

Students in higher education often face acute and chronic stressors in their everyday lives. Elevated stress levels have been associated with poor cognitive and academic outcomes [1]. Specifically, several studies have suggested acute stress negatively impacts executive function, a higher-order cognitive construct comprised of inhibitory control, working memory, and cognitive flexibility [2, 3] and memory [4, 5]. Acute stressors activate several physiological and psychological systems including the Hypothalamic Pituitary Adrenal and Sympathetic Adrenal Medullary axes, which release cortisol and catecholamines (e.g., epinephrine, norepinephrine) [6, 7]. Cortisol and catecholamines interact with receptors in brain regions such as the prefrontal cortex and hippocampus, which support executive function and memory [8, 9]. Importantly, the majority of acute stress and cognition studies utilize standardized lab-based cognitive tasks, however when examining acute stress and learning, it is important to model real-world learning contexts, such as lecture halls [5].

In higher education, lectures remain the most common content delivery format [10]. During lectures, the mind wanders from the task at hand toward internal information [11]. Mind wandering has been reported to occur 30–40% of the time during a lecture [12, 13]. Notably, increased mind wandering has been associated with reduced lecture comprehension [12, 14, 15]. With respect to factors that may modulate mind wandering, such as stress, several experimental studies have been conducted. For instance, Smallwood and colleagues (2009) [16] found that negative mood induction resulted in more mind wandering and less ability to disengage from task-irrelevant thoughts than positive mood. In an investigation examining the effects of acute stress on sustained attention and mind wandering, participants exposed to the Trier Social Stress Test (TSST), who reported high levels of negative mood, exhibited more mind wandering, as well as more variable reaction times and committed more errors on the Sustained Attention to Response Task [17]. Banks and colleagues (2015) found that individuals who were exposed to a writing task designed to induce psychological stress experienced increased mind wandering compared to individuals exposed to a neutral writing task [15]. Felt and colleagues (2021) posit that since acute stress has been associated with impaired executive function, this may lead to increased mind wandering. Furthermore, the authors suggest mind wandering may act as a mechanism to allow the individual to “distance” themselves from the acute stressor [18, 19].

When considering acute stressors and memory, a meta-analysis by Shields and colleagues (2017) suggests stress can either disrupt or improve aspects of memory depending on several factors. For instance, when stress occurred prior to or during encoding it impaired episodic memory except in instances where both the stressor-encoding delay was very brief and the study materials were relevant to the stressor [4]. On the other hand, stress improved episodic memory postencoding, except when the stressor occurred in a different environment from where studying occurred. In a narrative review by Vogel & Schwabe (2016) they highlight that stress may impede updating memories with new information, which is a critical element of the learning process. Taken together, there is evidence suggesting acute stress may increase mind wandering and impair memory, leading to less “durable” learning [20]. To date however, there is no direct evidence examining the role of acute stress on mind wandering during a lecture and subsequent performance on a lecture comprehension assessment. Thus, the present study aimed to examine the effects of acute stress on mind wandering during a twenty-minute video lecture and subsequent lecture comprehension assessment in young adults. We hypothesized that individuals exposed to acute stress would engage in more mind wandering and exhibit reduced lecture comprehension.

## Method

### Participants

As there were several variables of interest (i.e., mind wandering, lecture comprehension) an *a priori* sample size of 40 participants was generated from the smallest reported effect size of ηp2 = 0.05 [21], powered at 0.80, with an alpha of 0.05 using G*Power 3 software [22]. Forty participants (20 female, mean age = 22.3; SD = 2.6, age range = 18–28) from the Western University community were recruited via online advertisements and class presentations for this study (see Table 1). The recruitment period began August 8^th^ 2022 and concluded October 25^th^ 2022. Participants were excluded if they were: > 30 years of age, using tobacco, marijuana, or other recreational drugs [23, 24], taking prescription medications for chronic health conditions (e.g., cortisol-related disorders; [25, 26] taking prescription medications for depression or anxiety [27], pregnant and/or breastfeeding, diagnosed with a learning-related condition (i.e., ADHD, dyslexia), or experiencing fever or illness on the day of the experiment [28]. Further, participants were excluded if they had previously seen the lecture used in the experiment.

**Table 1:** Demographics.

	Stress	Control
N	20	20
Age	22.05 (2.6)	22.55 (2.6)
Sex (no. females)	8	12
Years of Education	16.37 (2.6)	17.10 (1.4)
GPA	3.68 (0.2)	3.74 (0.2)
Sleep Duration (h)	7.92 (0.7)	7.86 (1.5)
OC Use (no.)	2	2
Menstrual phase (no. in luteal phase)	3	1
GLTEQ Score	53.15 (27.5)	58.3 (28.5)
PSS	15.50 (7.4)	12.95 (5.2)
STAI-T	38.45 (10.1)	35.80 (9.0)

Note. Values reflect means and standard deviations in parentheses. no. = number of, OC use = Oral contraceptive use, GLTEQ: Godin Leisure Time Exercise Questionnaire, PSS = Perceived Stress Scale, STAI-T = State Trait Anxiety Inventory–Trait. Regarding gender, twenty participants identified as women and twenty participants identified as men.

Participants provided informed written consent of a protocol approved by the Health Sciences Research Ethics Board (#121155) at Western University and this study was conducted in accordance with the most recent iteration of the Declaration of Helsinki. We report how we determined our sample size, all data exclusions (if any), all manipulations, and all measures in the study.

### Materials

#### Lecture

Participants watched a twenty-minute video lecture narrated by a university professor. Video lectures of a similar duration (i.e., twenty-one minutes) have been used in prior mind wandering studies [12]. The video lecture covered food-borne illness and was selected from a series of publicly available lectures on the “Classes Without Quizzes” subchannel on YouTube. Video lectures from this channel have been used in prior lecture-based studies [29]. Participants were not permitted to take notes during the lecture.

#### Stress induction

The Trier Social Stress Test (TSST) has been used in numerous studies to induce acute physiological and psychological stress responses [30, 31]. Participants were instructed that they would engage in a five-minute speech task where they would describe why they would be a good candidate for their ideal job to a two-judge panel. Participants were then instructed “Your speech will be videotaped and reviewed by a panel of judges trained in public speaking”. Participants were then left alone in the room for ten minutes to prepare their speech. Participants then delivered their five-minute speech to the same two-judge panel. Following the speech, participants were then instructed to perform a math task constituting of serial subtractions of thirteen from 1022 (i.e., 1022 minus 13 followed by 1009 minus 13 etc.,) and instructed if a mistake is made, they must begin subtractions again from 1022. The math-task lasted for five minutes. A “dummy” camera was set up in front of the participant but was not filming, see [32] for a detailed review of the TSST protocol.

### Measures

#### Demographic information and participant questionnaires

Age, sex, gender, years of education (e.g., first year undergraduate student), self-reported grade point average (GPA), bedtime, wake-up time, oral contraceptive use, and menstrual cycle phase (i.e., follicular, luteal) were collected. The Godin Leisure Time Exercise Questionnaire (GLTEQ; [33] was used as a measure of weekly self-reported exercise engagement, with a greater score reflecting greater weekly exercise engagement. The Perceived Stress Scale (PSS; [34] was used to assess perceived stress in the past month. The PSS is comprised of ten items, which assess the degree to which situations in one’s life are appraised as stressful, with a greater score indicating greater perceived stress. The computed Cronbach’s α for the PSS was α = 0.89.

#### State trait anxiety

The State Trait Anxiety Inventory (STAI; [35]) was used to measure state and trait anxiety. Twenty items assessed state anxiety (STAI-S) and twenty items assessed trait anxiety (STAI-T), with a greater score indicating greater levels of anxiety. The computed Cronbach’s α for STAI-S and STAI-T were α = 0.94 and α = 0.91 respectively.

#### Heart rate

Heart rate was assessed via a chest-strap heart rate monitor (Polar H10 Wearlink + Coded Transmitter, Polar Electro Inc., Lake Success, NY, USA) in beats per minute (bpm).

#### Blood pressure

Blood pressure was assessed via an electronic blood pressure cuff (Omron BP7455CAN, Omron Healthcare Co., Kyoto, Japan) in millimetre of mercury (mmHg).

#### Salivary cortisol

Participants provided saliva samples (∼ 0.5 mL) using a passive drool method. Immediately after collection, the saliva vials were stored in a– 80° C freezer until assayed in duplicate using a high sensitivity enzyme immunoassay (Salimetrics LLC, Carlsbad, CA) according to manufacturer instructions. The intra-assay CV was 4.6% and the inter-assay CV was 6.00%. Sensitivity for these assays was 0.007 μg/dL. Cortisol concentrations were converted from μg/dL to nmol/L for consistency with previously published works in human stress literature.

#### Mind wandering

Mind wandering was assessed via a validated tool utilized in university-based classes by Wammes and colleagues (2016) [13]. Three mind wandering assessments (i.e., MW1, MW2, and MW3) were distributed equally throughout the lecture, as conducted in prior work [36]. MW1 was presented at approximately the seventh minute, MW2 was presented approximately at the fourteenth minute, and MW3 was presented approximately at the nineteenth minute. The material pertinent to the quiz questions was presented within 1–3 minutes before each probe. The screen provided the following instruction: “Which of the following responses best characterizes your mental state just before this screen appeared?”, with three response options: on task, intentionally mind wandering, or unintentionally mind wandering.

#### Lecture comprehension

Lecture comprehension was assessed via a 15-question paper and pencil assessment comprising of eight multiple choice questions and seven fill-in-the-blank questions.

### Procedure

**Fig 1:**
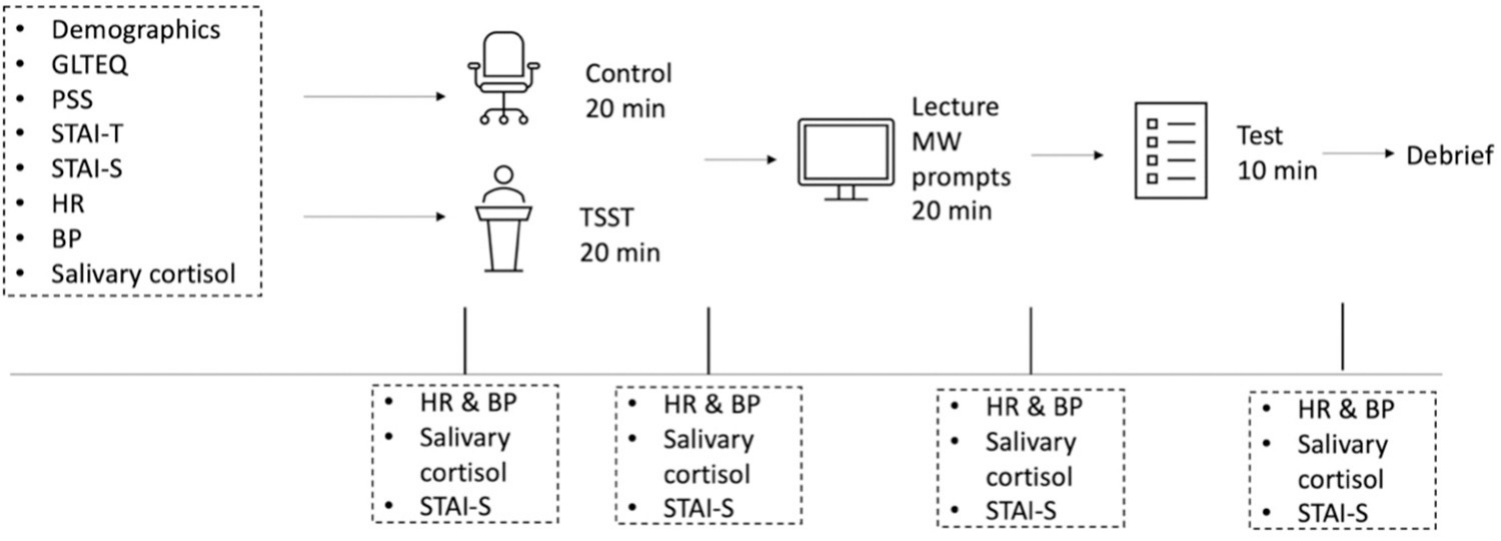
Study schematic.

Participants abstained from exercise and caffeine 3 h prior to lab arrival and food or beverage consumption (except water) for 1 h prior to lab arrival [37]. Participants also abstained from dental work the day prior and brushing teeth for 1 h prior to lab arrival. All study procedures were completed between 12:00 to 6:00 pm to minimize circadian variation in salivary cortisol [38]. Participants were randomly assigned to a Control (n = 20) or Stress (n = 20) condition using the online randomizer tool (random.org). See Fig 1 for a full study schematic.

Upon entry into the lab, participants were seated and filled out the demographic questionnaire, GLTEQ, PSS, STAI-T, and STAI-S. Blood pressure was assessed twice in a seated position and participants were fitted with a heart rate monitor to provide continuous HR measurement. Participants were instructed on how to passively drool and provided their first saliva sample. These baseline assessments took approximately 30 minutes which promoted acclimation to the lab environment prior to the Control or Stress condition [3]. HR, BP, state anxiety, and a saliva sample were collected again prior to either condition. Participants in the Control condition remained in a seated position for 20 minutes in the lab with the experimenter and were not permitted to use technology (i.e., smartphones or laptops) during this time. Participants in the Stress condition were exposed to the TSST in a separate room in the lab.

Immediately following either condition (i.e., Control, Stress), HR, BP, state anxiety, and a saliva sample were collected. Participants were then instructed that: they would watch a twenty- minute video lecture, three prompts would appear during the lecture in which they would need to respond regarding their current mental state, and that a lecture comprehension assessment would follow. Participants were not permitted to take notes during the lecture. Immediately following the lecture, HR, BP, state anxiety, and a saliva sample were collected. Participants were then presented with a paper and pencil lecture comprehension assessment and instructed they had 10 minutes to complete the assessment. Immediately following the lecture comprehension assessment, HR, BP, state anxiety, and a final saliva sample were collected. Participants were then debriefed regarding the true purpose of the study. Study data are available in the Supplementary files, while code for the lecture video with embedded mind wandering prompts is openly available on GitHub (https://github.com/Morava83/DataCollection).

## Results

### Statistical analyses

All variables were assessed for normality and linearity, where appropriate. Greenhouse-Geisser corrections for violations of sphericity are reported where appropriate (corrected degrees of freedom reported to one decimal place) and an alpha level of 0.05 was used for all ANOVA models. Effect sizes (i.e., Cohen’s d, Partial eta-squared) are reported where appropriate. Boxplots for dependent variables were constructed and inspected to screen for potential outliers to winsorize (Q3 + 1.5* Interquartile range or Q1–1.5*Interquartile range). All analyses were conducted with SPSS Version 28 and JASP Version 0.17.1.

HR, BP, and STAI-S scores were assessed via separate repeated mixed-model ANOVAs with a between-subject factor of Group (i.e., Control, Stress) and a within-subject factor of time. As salivary cortisol data exhibited deviation from normality, a log-transformation was applied to reduce skewness. Overall cortisol secretion was calculated via the “area under curve with respect to ground” (AUC_g_; [39]. Cortisol reactivity was calculated as the difference between the pre-TSST sample and the participant’s cortisol peak (i.e., maximum) sample [40]. Cortisol metrics were assessed using separate one-way ANOVAs with a between-subject factor of Group (i.e., Control, Stress). Cortisol metrics are presented for the whole sample, see Supplementary data for information regarding cortisol-responders and non-responders as defined by [41].

To assess mind wandering, unintentional and intentional mind wandering were collapsed for analyses (i.e., “on-task” coded as 1, “off-task” coded as 0) which is a technique used in prior investigations [36] and a binary logistic regression was conducted. Lecture comprehension scores were assessed using a one-way ANOVA with a between-subject factor of Group (i.e., Control, Stress). Bivariate correlations were used to assess the strength of the associations between stress measures (i.e., HR, STAI-S, and Salivary cortisol), mind wandering, and lecture comprehension.

### Group equivalency

Participants assigned to the Stress group versus Control group did not reliably differ with respect to age, sex, education, GPA, sleep duration, oral contraceptive use, menstrual phase, self-reported exercise engagement (i.e., GLTEQ score), perceived stress (i.e., PSS), trait anxiety (i.e., STAI-T), baseline HR, baseline SBP, baseline DBP, and pre-TSST cortisol (all *p*’s > 0.05).

### Stress response analyses

#### HR

**Fig 2:**
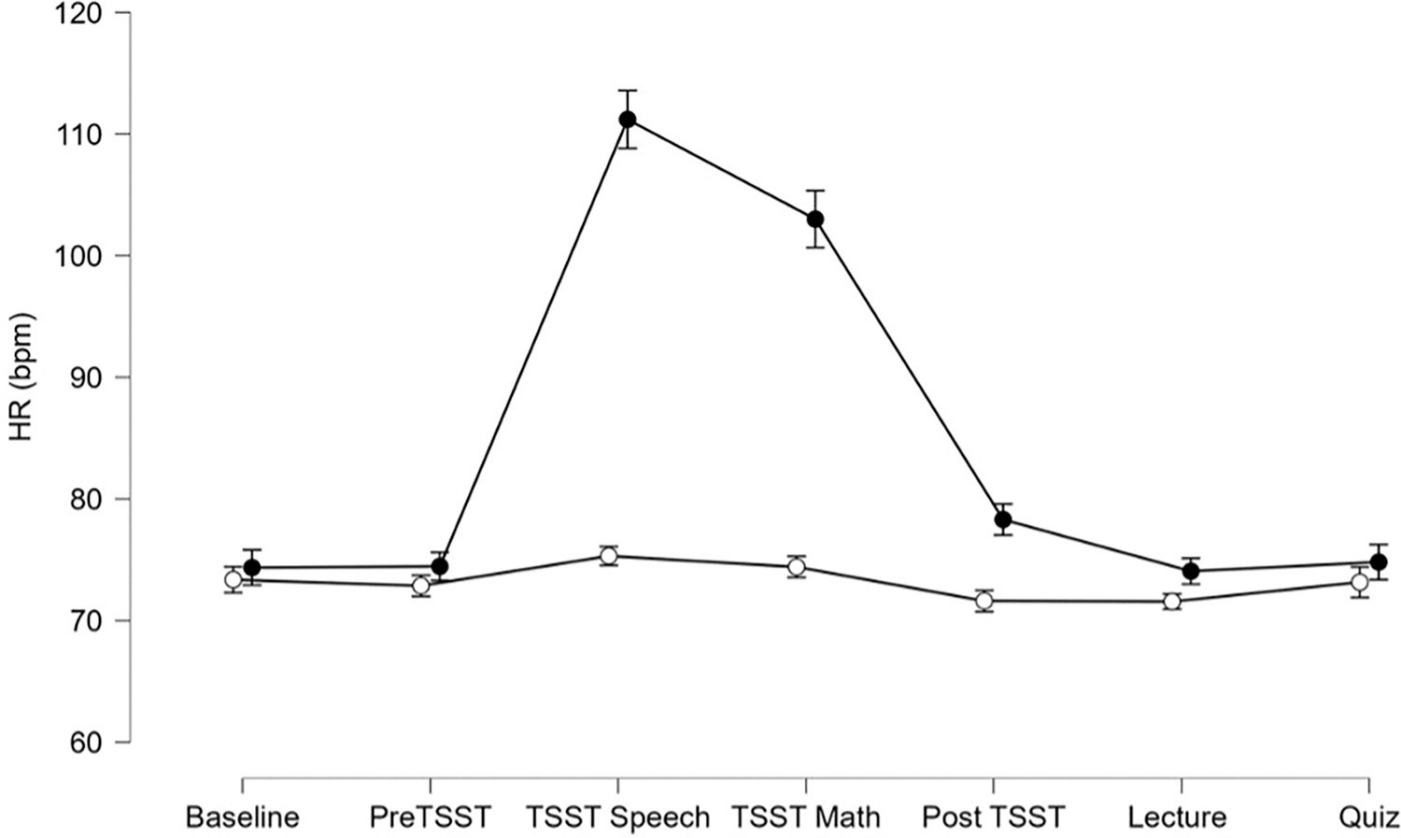
Heart rate (HR) in beats per minute (bpm) are depicted for the control group (open circles) and the Stress group (black circles). Error bars represent ± SEM.

Results yielded a significant Group by Time interaction for HR, F(3.5, 133.4) = 59.4, p < 0.001, ηp^2^ = 0.61. Simple main effects indicated HR was significantly elevated for the Stress group during the TSST Speech, F(1, 38) = 53.2, p < 0.001, ηp^2^ = 0.58 and TSST Math, F(1, 38) = 37.3, p < 0.001, ηp^2^ = 0.50, see Fig 2.

#### BP

Results yielded no significant main effects of Group, Time, or a Group by Time interaction for SBP, F(1, 38) = 1.48, p = 0.23, ηp^2^ = 0.04; F(3.0, 114.2) = 1.40, p = 0.24, ηp^2^ = 0.04, and F(3.0, 114.2) < 1, p = 0.49, ηp^2^ = 0.02. Results also yielded no significant main effects of Group, Time, or a Group by Time interaction for DBP, F(1, 38) < 1, p = 0.69, ηp^2^ = 0.04; F(3.1,117.8) = 2.27, p = 0.06, ηp^2^ = 0.06; F(3.1, 117.8) < 1, p = 0.67, ηp^2^ = 0.01.

#### STAI-S

**Fig 3:**
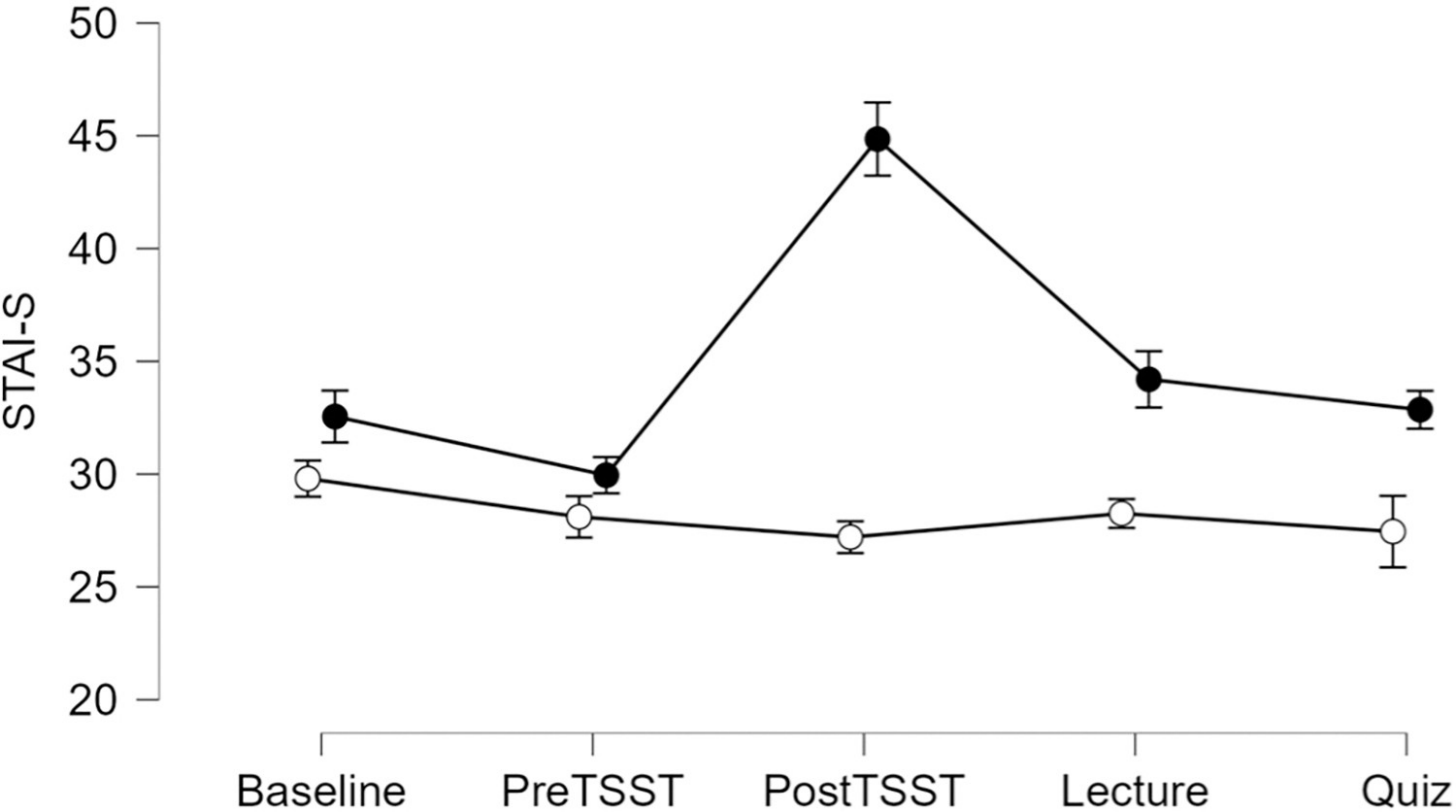
State anxiety scores are depicted for the control group (open circles) and the stress group (black circles). Error bars represent ± SEM.

Results yielded a significant Group by Time interaction for STAI-S, F(2.7, 103.7) = 21.1, p < 0.001, ηp^2^ = 0.36. Simple main effects indicated STAI-S was elevated for the Stress group immediately after the TSST, F(1, 38) = 37.0, p < 0.001, ηp^2^ = 0.49 and post-lecture, F(1,38) = 5.4, p = 0.02, ηp^2^ = 0.13, See Fig 3.

#### Cortisol

One participant yielded physiologically implausible values of cortisol (>100 nmol/l) and was removed from cortisol-based analyses only.

#### Overall cortisol output

**Fig 4:**
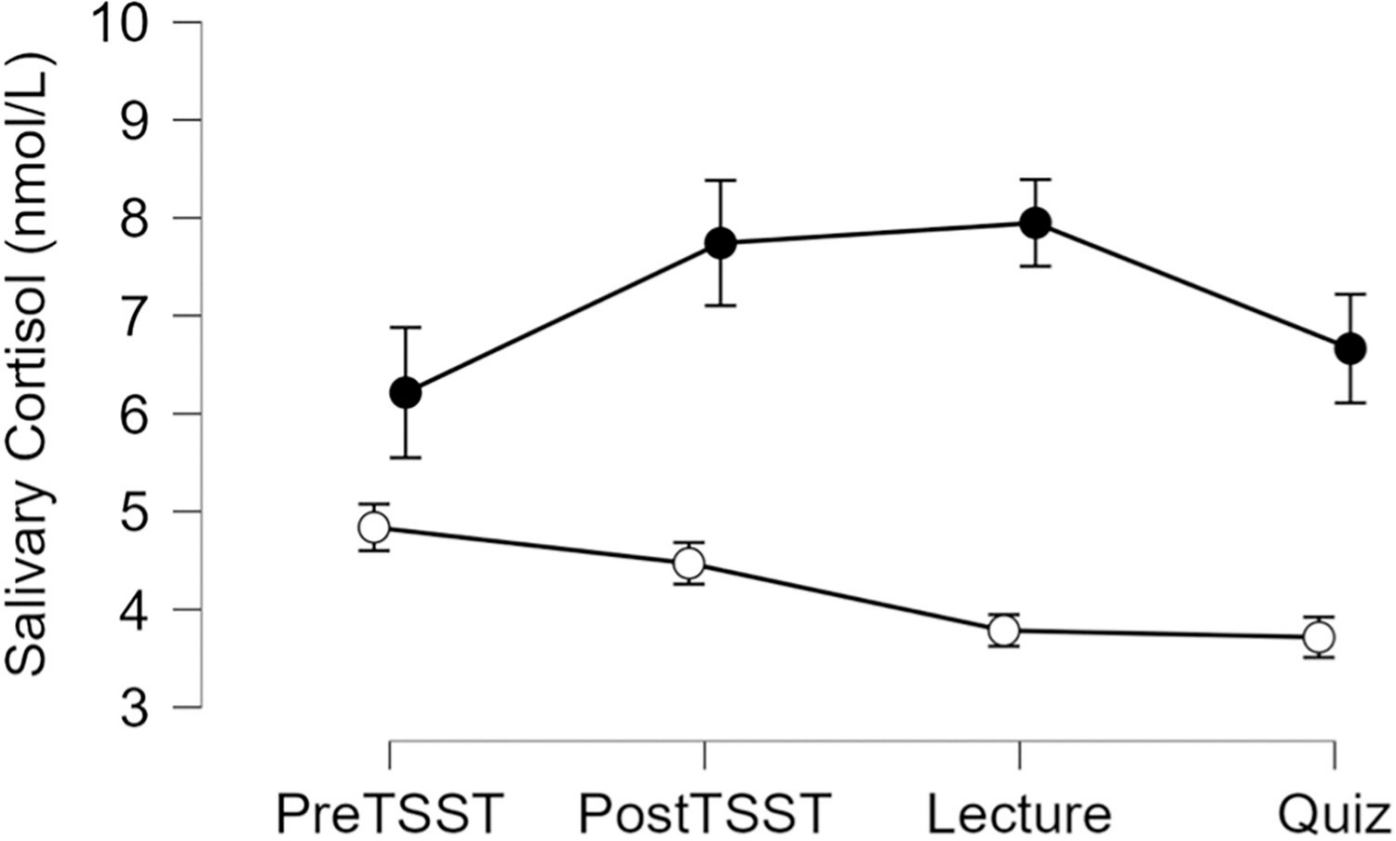
Raw salivary cortisol concentrations in nmol/L are depicted for the control group (open circles) and the stress group (black circles). Error bars represent ± SEM.

A one-way ANOVA indicated significant differences in overall cortisol output between the Stress and Control groups, F(1, 37) = 8.93, p = 0.005, ηp^2^ = 0.19, such that the Stress group demonstrated greater overall cortisol output than the Control group, see Fig 4.

#### Cortisol reactivity

A one-way ANOVA indicated significant differences in cortisol reactivity between the Stress and Control groups, F(1, 37) = 8.0, p = 0.008, ηp^2^ = 0.18, such that the Stress group demonstrated higher cortisol reactivity than the Control group, see Fig 4.

#### Mind wandering

The rates for each probe reponse (i.e., on task, unintentionally MW, or intentionally MW) for each checkpoint are presented below (Table 2). Notably, the response rates for on task, unintentional, and intentional MW did not significantly differ by group at any checkpoint based on Chi-squared tests, all *p*’s < 0.05 (See Supplementary files for further details).

**Table 2:** Probe response rates at each lecture checkpoint.

	MW1	MW2	MW3
On Task	23	27	20
Unintentionally MW	12	10	17
Intentionally MW	5	3	3

Binary logistic regressions at each time point (i.e., MW1, MW2, and MW3) indicated there were only significant group differences at the first time point (i.e., MW1). Group differences indicated the Stress group engaged in more mind wandering (i.e., off-task behavior) than the Control group, OR = 4.5, 95% CI = 1.2, 17.4, p = 0.03.

#### Lecture comprehension

**Fig 5:**
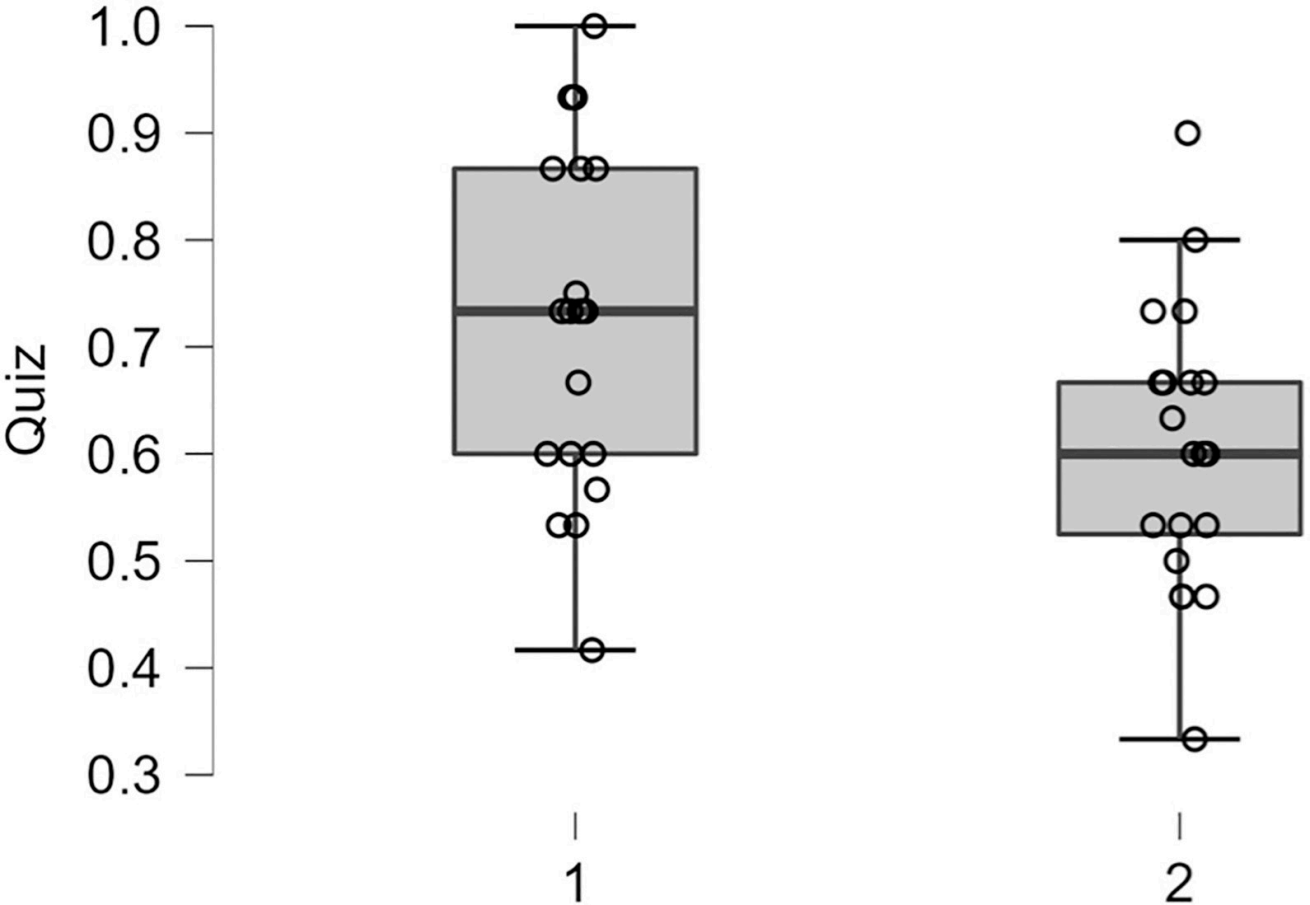
Boxplots represent the lecture comprehension scores for the Control (1) group and Stress (2) group.

A one-way ANOVA indicated significant differences in lecture comprehension performance between the Stress and Control groups, F(1, 38) = 6.29, p = 0.02, ηp^2^ = 0.14, such that the Control group reported improved lecture comprehension performance (72%) than the Stress group (60%), see Fig 5. A one-way ANOVA indicated no significant differences in lecture comprehension performance for material presented around the MW probes between the Stress and Control groups, F(1, 38) = 2.40, p = 0.13, ηp^2^ = 0.06.

#### Associations between stress measures, mind wandering, and lecture comprehension

*Stress measures*. When comparing associations between different stress measures, Pearson’s correlations (Table 3) revealed that HR during the TSST (Speech) and TSST (Math) were significantly positively correlated with cortisol metrics (i.e., Cortisol reactivity, AUCg) and STAI-S scores immediately post TSST.

**Table 3:** Pearson’s correlations between physiological, psychological, and learning related variables.

Variable		TSST_SHR		TSST_MHR		TSST_SBP	TSST_DBP	Cort_Reactivity		AUCg	MW1			MW2		MW3		Quiz		STAIPostTSST		STAIPostLec
1. TSST_SHR	Pearson’s r	—																				
	p-value	—																				
2. TSST_MHR	Pearson’s r	0.919	***	—																		
	p-value	< .001		—																		
3. TSST_SBP	Pearson’s r	0.014		0.018		—																
	p-value	0.933		0.913		—																
4. TSST_DBP	Pearson’s r	0.268		0.362	*	0.164	—															
	p-value	0.099		0.024		0.318	—															
5. Cort_Reactivity	Pearson’s r	0.445	**	0.414	**	-0.070	0.027	—														
	p-value	0.005		0.009		0.670	0.869	—														
6. AUCg	Pearson’s r	0.579	***	0.466	**	0.102	0.008	0.454	**	—												
	p-value	< .001		0.003		0.536	0.962	0.004		—												
7. MW1	Pearson’s r	0.208		0.187		-0.034	-0.187	0.127		0.310		—										
	p-value	0.204		0.254		0.835	0.254	0.447		0.058		—										
8. MW2	Pearson’s r	0.127		0.220		-0.114	0.094	0.395	*	0.120		0.074		—								
	p-value	0.441		0.178		0.488	0.570	0.014		0.474		0.656		—								
9. MW3	Pearson’s r	0.184		0.274		-0.110	0.066	0.266		0.096		0.187		0.580	***	—						
	p-value	0.261		0.092		0.507	0.689	0.107		0.568		0.254		< .001		—						
10. Quiz	Pearson’s r	-0.269		-0.225		0.075	-0.173	-0.174		-0.193		-0.262		-0.270		-0.416	**	—				
	p-value	0.097		0.169		0.648	0.293	0.296		0.246		0.107		0.096		0.009		—				
11. STAIPostTSST	Pearson’s r	0.489	**	0.406	*	-0.172	0.012	0.226		0.282		0.337	*	0.322	*	0.283		-0.396	*	—		
	p-value	0.002		0.010		0.296	0.944	0.172		0.086		0.036		0.046		0.081		0.013		—		
12. STAIPostLec	Pearson’s r	0.211		0.128		0.057	0.188	-0.037		0.046		0.177		0.259		0.114		-0.426	**	0.723	***	—
	p-value	0.197		0.437		0.730	0.251	0.826		0.786		0.282		0.111		0.491		0.007		< .001		—

* p < .05

** p < .01

*** p < .001

*Stress measures and mind wandering*. Point-biserial correlations (Table 3) revealed that STAI-S scores immediately post TSST were significantly positively correlated with mind wandering at the first and second time point (i.e., MW1 and MW2), while cortisol reactivity was significantly positively correlated at only the second time point (i.e., MW2).

*Stress measures and lecture comprehension*. Pearson’s correlations (Table 3) revealed the only STAI-S scores immediately post TSST and post lecture were significantly negatively correlated with lecture comprehension.

*Mind wandering and lecture comprehension*. Point-biserial correlations (Table 3) revealed only mind wandering at the third time point (i.e., MW3) was significantly negatively correlated with lecture comprehension. Point-biserial correlations (Table 4) revealed mind wandering at the second and third time points (i.e., MW2, MW3) were significantly negatively correlated with lecture comprehension performance on material presented around the MW probes.

**Table 4:** Pearson’s correlations between responses on mind wandering probes and lecture comprehension performance on material presented around the MW probes.

Variable		AroundProbe		MW1	MW2		MW3
1. AroundProbe	Pearson’s r	—					
	p-value	—					
2. MW1	Pearson’s r	-0.205		—			
	p-value	0.205		—			
3. MW2	Pearson’s r	-0.450	**	0.051	—		
	p-value	0.004		0.753	—		
4. MW3	Pearson’s r	-0.426	**	0.152	0.587	***	—
	p-value	0.006		0.350	< .001		—

* p < .05

** p < .01

*** p < .001

## Discussion

The present study examined the effects of acute stress on mind wandering during a video lecture and subsequent lecture comprehension. Individuals exposed to acute stress via the TSST endorsed greater mind wandering at the first checkpoint and lower lecture comprehension scores. Furthermore, state anxiety post TSST was positively associated with mind wandering at the first and second checkpoint and negatively associated with lecture comprehension. Only mind wandering at the third checkpoint was negatively associated with overall lecture comprehension scores, while mind wandering at the second and third checkpoint were negatively associated with lecture comprehension of material presented around the probes. Several issues within these overarching findings warrant commentary.

### The impairing effects of acute stress on mind wandering and lecture comprehension

Our stress induction via the TSST was effective as evidenced by significant increases in HR, salivary cortisol, and state anxiety in exposed individuals. In line with prior studies, acute stress increased mind wandering [15, 17], however this increase was only present at the first checkpoint in the video lecture. Notably, Vinski and colleagues (2012) also found the increase in mind wandering was transient [17]. One plausible explanation is the proximity between the TSST and the first mind wandering checkpoint. In accordance with the current concerns hypothesis [11, 42], the “current concerns” of one’s TSST performance may be more salient than the external environment (i.e., video lecture), particularly right after the stressor, leading to off-task behavior. Alternatively, the Control Failure x Concerns model by McVay & Kane (2010) posits that mind wandering represents an executive control failure that is determined by the presence of “automatically generated thoughts” (i.e., the current concerns from the TSST environment) and the ability of executive function systems to address interference [43]. As there is evidence that acute stress negatively impacts executive function [4], this represents a potential mechanism underpinning the relationship between acute stress and increased mind wandering.

Regarding acute stress and lecture comprehension, the Stress group demonstrated lower performance than the Control group. This finding is in line with a meta-analysis which found acute stress occurring prior to encoding impaired episodic memory [4]. Notably, several groups [4, 44] have highlighted the interaction between the time course of the stress response and memory processes (e.g., encoding, retrieval) uniquely impact the effects of acute stress on memory. For instance, when examining the effects of stress on encoding, stress-encoding delay (i.e., the delay in minutes between stress onset and encoding) and whether the study items were relevant to the stressor were moderators. A greater stress-encoding delay resulted in impairing effects, particularly at a delay of approximately 22 minutes, as well as when study materials were not relevant to the stressor [4]. In our study, the video lecture was administered approximately 25 minutes post TSST start and the study materials were regarding food-borne illness, which do not pertain to the TSST. Importantly, as the majority of studies examining acute stress and memory utilize standardized memory assessments, our study is the first to provide direct evidence of negative effects of acute stress on memory pertaining to information presented in a video lecture format.

### State anxiety uniquely affects mind wandering and lecture comprehension

Our study examined stress using a multidimensional approach, as recommended by prior work [45, 46] and found robust elevations to all acute stress measures, except BP, as a result of the TSST. The lack of elevation to BP in this investigation may be due to measurement, as BP was measured in a non-continuous manner. There were positive correlations between physiological (i.e., HR, cortisol) and psychological indices (i.e., STAI scores) of stress. Notably, state anxiety was negatively associated with both mind wandering at the first and second checkpoints and with lecture comprehension scores. This is an important finding, as it indicates that the perception of elevated stress, rather than physiological elevations may be more associated with processes such as mind wandering and subsequent lecture comprehension. This may be in part due to cognitive resources being utilized to process the psychological experience of stress, leading to off-task behavior and reduced lecture comprehension. For instance, prior work examining individuals who experience math anxiety—“an adverse emotional reaction to math or the prospect of doing math” [47] has indicated negative effects on working memory and other cognitive domains [48, 49]. Alongside the potential changes in cognitive resource allocation, it is important to note that state anxiety remained elevated post lecture, suggesting that perhaps the persistence of elevated state anxiety may have disrupted the learning process during the lecture, impacting downstream comprehension.

### The relationship between mind wandering and lecture comprehension

Regarding the relationship between mind wandering and lecture comprehension, prior literature has suggested (1) as time on a given task increases, mind wandering increases [11, 15, 43], with notable exception [13] and (2) increased mind wandering is associated with decreased lecture comprehension [12, 14, 15]. Descriptively, our findings suggest irrespective of group, the percentage of individuals reporting mind wandering was greater at the third checkpoint than at the first and second checkpoints, as well as that for individuals who were mind wandering, the majority of participants were unintentionally mind wandering at all checkpoints. When examining correlations between responses on mind wandering probes and performance on material presented around the probes, we found negative correlations of moderate strength at the second and third checkpoints, which is congruent with findings in the literature with respect to time of task effects.

Our correlational findings are in line with prior studies such as Risko and colleagues (2012) who reported negative correlations between mind wandering and test performance [50]. Notably, Risko and colleagues (2012) found that correlations between mind wandering and test performance may vary depending on lecture content, as the authors presented a Psychology, Economics, and Classics lecture and reported correlations between mind wandering and test performance varying from r = - 0.03 to—0.50 [50]. Previous research examining vigilance tasks have suggested increasing time on task is associated with negative affective states that could reduce participant motivation to orient attention toward the task, which may explain in part the association between increased mind wandering at the third checkpoint and reduced lecture comprehension [51]. Alternatively, the negative correlation between mind wandering and lecture comprehension at the third checkpoint may be in part due the amount or nature of the content in the latter third of the lecture. Taken together, we have provided evidence to suggest that acute stress may impact lecture comprehension directly and perhaps in a limited capacity indirectly through mind wandering, although further examination of this mechanistic pathways is warranted.

### Study limitations and future directions

Notable study strengths include our multidimensional approach of examining acute stress and modelling a real-world learning environment through a video lecture and subsequent comprehension test. Limitations include the sample size, particularly for investigating the robustness of our correlations, non-continuous BP assessment, and that the lecture comprehension assessment was conducted immediately after the lecture so we are unable to comment on the effects of stress on delayed recall. Second, although mind wandering probes are frequently used to examine on-task behavior, they have inherent limitations such as only capturing a “moment” of behavior, as well as having binary response options (i.e., on-task or off-task). In addition, the number of probes presented during a task may provide varying insights on mind wandering patterns. Lastly, we did not assess the content of thoughts participants were having when mind wandering so we cannot determine whether they were thinking about the stressor or other task-unrelated thoughts during the lecture. Future studies should examine methods for students to either “buffer” or cope with acute stress to promote durable learning.

## Conclusion

Our findings demonstrate that acute stress increased mind wandering at the first checkpoint during a twenty-minute video lecture and negatively impacted lecture comprehension in young adults. Furthermore, state anxiety was positively associated with mind wandering at two checkpoints during the lecture and negatively associated with lecture comprehension. Lastly, mind wandering at the second and third checkpoint was negatively associated with lecture comprehension material presented around the probes.

## Supporting information

S1 File Lecture comprehension questions. (DOCX)

S2 File Mind wandering tables. (DOCX)

S1 Data Full data. (XLSX)
