# Supplementary material for: Acute stress negatively impacts on-task behavior and lecture comprehension
Source: PLoS One. 2024 Feb 6;19(2):e0297711. doi: 10.1371/journal.pone.0297711 (PMC10846713; doi:10.1371/journal.pone.0297711)
Supplement: S1 File — (DOCX) [file pone.0297711.s001.docx]

Lecture Comprehension Questions

**Multiple Choice**

*1. Which of the following statements is true?*

a) Bacteria cannot live (grow and multiply) on their own.

b) Virus and fungi can live (grow and multiply) on their own.

c) Bacteria and Virus are the same thing.

d) Fungi cannot live (grow and multiply) on their own.

e) Virus cannot live (grow and multiply) on their own.

f) Fungi and Virus are the same thing.

*2. Which of the following statements is true?*

a) In low-income countries benefits of small scale production and distribution are offset by the lack of hygiene and enforced regulations.

b) In high-income countries benefits of small scale production and distribution are offset by the lack of hygiene and enforced regulations.

c) In low-income countries the dangers of small scale production and distribution are offset by the lack of hygiene and enforced regulations.

d) In high-income countries the dangers of small scale production and distribution are offset by the lack of hygiene and enforced regulations.

e) In low-income countries benefits of small scale production and distribution are offset by the great of hygiene and enforced regulations.

f) In medium-income countries benefits of small scale production and distribution are offset by the great of hygiene and enforced regulations.

3. *Contamination of food can occur during…*

a) Production (field and feed).

b) Processing (slaughter and slice).

c) Serving (cook and kitchen).

d) All of the above

e) 2 of the above

f) None of the above

*4. Food poisoning…*

a) Occurs when food is the vehicle for the ingestion of a microbial organism, which may then establish itself in the host and cause disease, either by a toxin or by invasion.

b) Is from consuming something, which may be food, contaminated by toxins, which could be chemical or microbial in origin.

c) Is more common than food associated infections.

d) Symptoms occur after days to weeks.

e) As a form of murder, is at present, easy to get away with.

f) None of the above

*5. Which of the following statements is true?*

a) Our food is very unsafe.

b) Ground beef can safely be eaten raw.

c) Our food is generally safe.

d) Listeria is fatal 100% of the time.

e) You will never get sick by eating raw oysters.

f) Salmonella cannot be found in eggs.

*6. Which pre-formed toxin is not associated with food?*

a) Clostridium

b) Tetanus

c) Staphylococcus

e) Bacillus

f) None of the above

*7. Which bacteria is associated with dairy and cream products?*

a) Clostridium

b) Tetanus

c) Staphylococcus

e) Bacillus

f) None of the above

8. *Which of the following statements is true?*

a) Listeria is a virus

b) E coli is a virus

c) Listeria is a bacteria

d) A bacteria cannot live on its own.

e) A virus can live on its own.

f) None of the above

Fill In The Blanks

1. For every disease sample, approximately 100 people actually had the disease but were never reported.

2. In the USA, food-associated illness causes 5000 deaths.

3. In high income countries, food-associated illnesses are most commonly acquired from catering facilities.

4. Today’s food travels approximately 2000 km.

5. Antibiotics are used to improve weight gain in animals.

6. The most recent outbreak of infant botulism was associated with unpasteurized honey.

7. Ergot poisoning causes a spasm of the arteries.
