# Supplementary material for: Acute stress negatively impacts on-task behavior and lecture comprehension
Source: PLoS One. 2024 Feb 6;19(2):e0297711. doi: 10.1371/journal.pone.0297711 (PMC10846713; doi:10.1371/journal.pone.0297711)

1. Contingency tables depicting on task responses, unintentional, and intentional mind wandering responses at the three mind wandering checkpoints during the lecture.

1 = On Task, 2 = Unintentionally Mind Wandering, 3 = Intentionally Mind Wandering


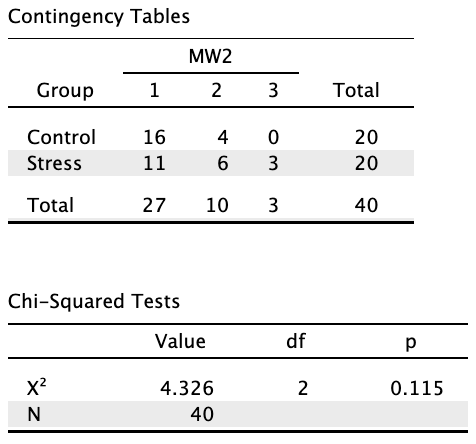

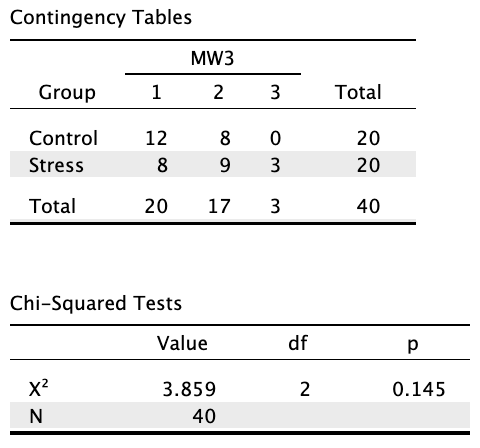

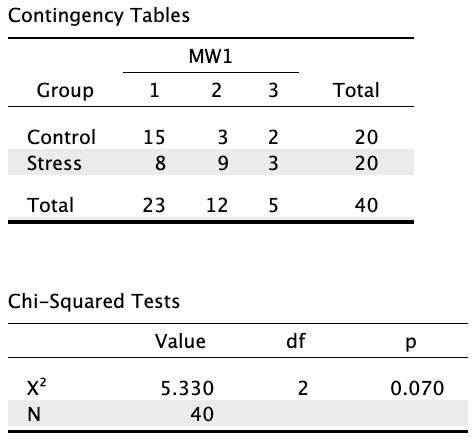


1. Point biserial correlations between the type of mind wandering (i.e., intentional versus unintentional) and total lecture comprehension scores.


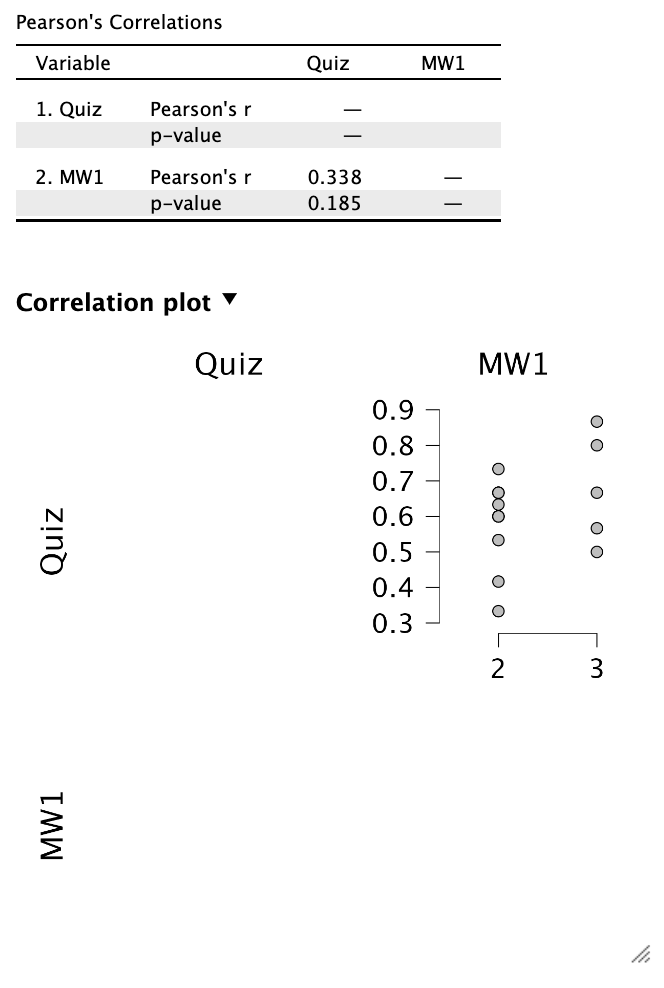

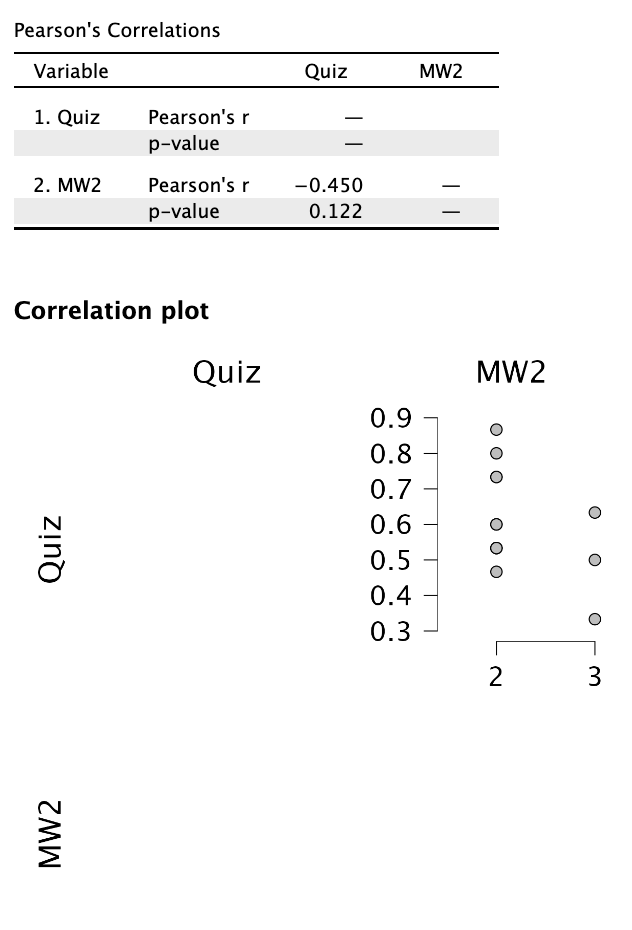

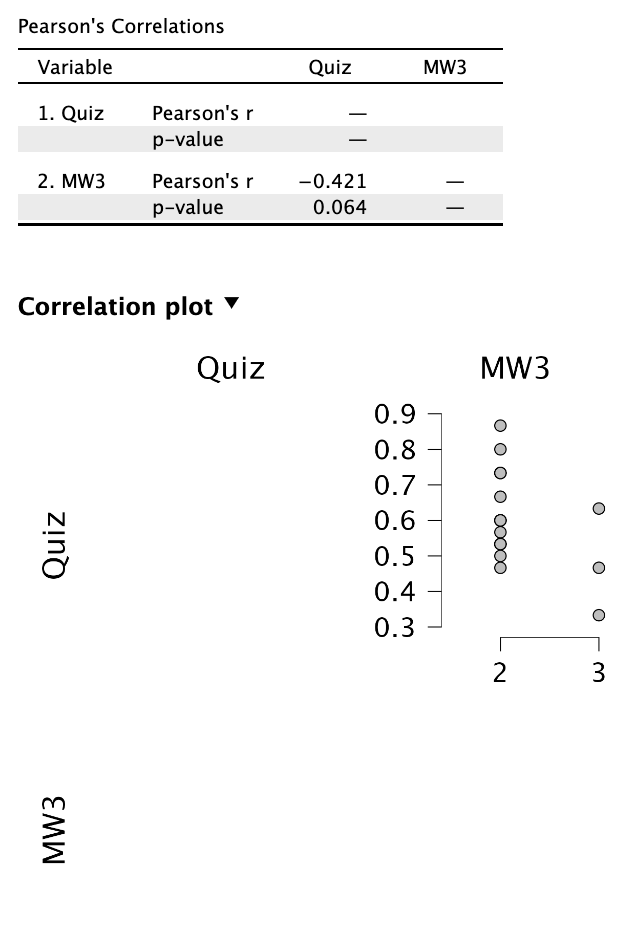


1. Point biserial correlations between the type of mind wandering (i.e., intentional versus unintentional) and around the probe comprehension scores.


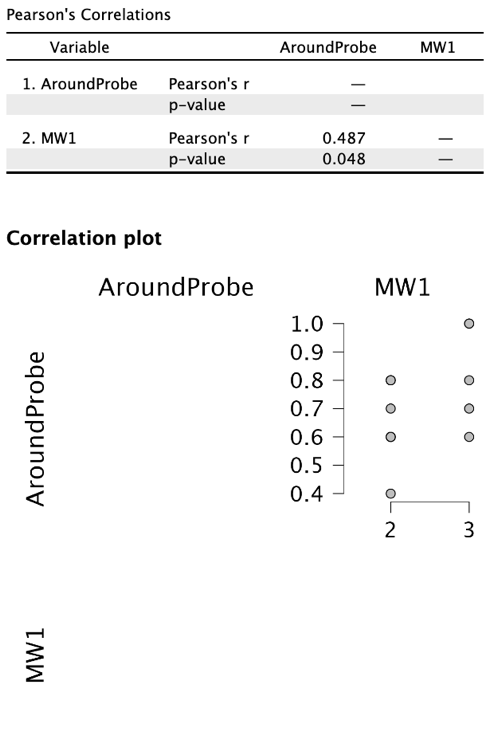

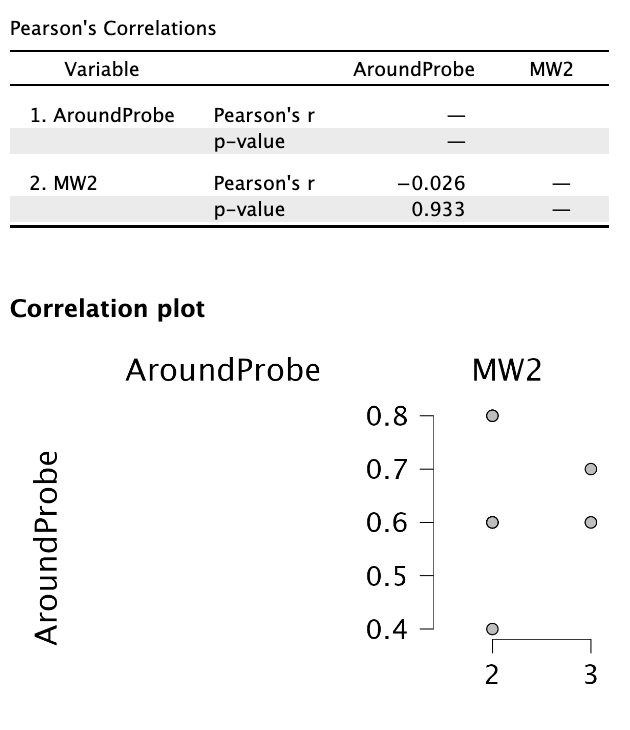

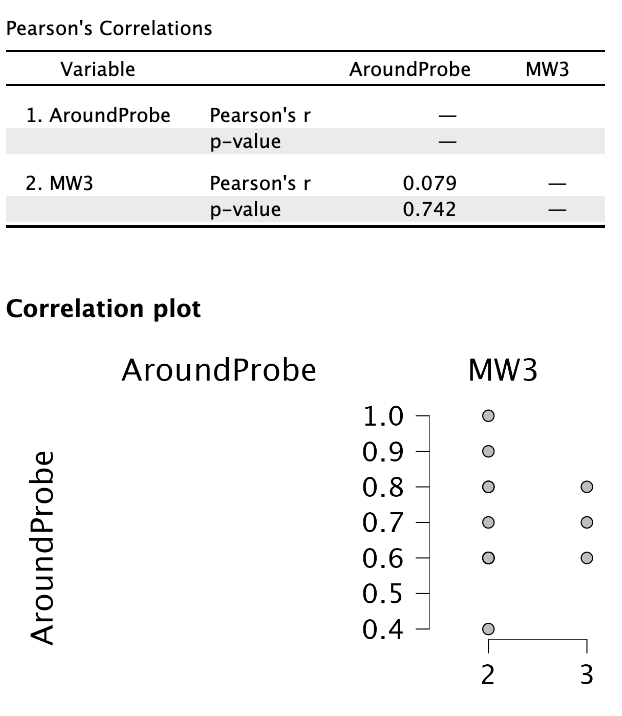

Supplement: S2 File — (DOCX) [file pone.0297711.s002.docx]
